# Drink Choice is Important: Beverages Make a Substantial Contribution to Energy, Sugar, Calcium and Vitamin C Intake among Australians

**DOI:** 10.3390/nu11061389

**Published:** 2019-06-20

**Authors:** Malcolm D. Riley, Gilly A. Hendrie, Danielle L. Baird

**Affiliations:** Commonwealth Scientific and Industrial Research Organisation (CSIRO) Health and Biosecurity, P.O. Box 10041, Adelaide BC, SA 5000, Australia; Gilly.Hendrie@csiro.au (G.A.H.); Danielle.Baird@csiro.au (D.L.B.)

**Keywords:** beverage intake, nutrient intake, Australian dietary survey, dietary energy intake, calcium intake, vitamin C intake, sugar intake, adults, children

## Abstract

It is important to understand the role of beverages in population dietary intake in order to give relevant advice. Population estimates were derived from one-day food recall dietary data from 12,153 participants in the 2011–2012 Australian National Nutrition and Physical Activity Survey. Almost all Australians (99.9%) consumed at least one beverage on the day of the survey, accounting for 16.6% of the total energy intake for adults (aged 19 years and over) and 13.0% for children (aged 2–18 years). Similarly, beverages contributed 26–29% to calcium intake, 22–28% to vitamin C intake, and 35–36% to sugar intake. Water was consumed on the day of the survey by 84.1% of Australian adults and 90.5% of children. For adults, the greatest beverage contributors to total energy intake were alcoholic drinks (5.6%), coffee (3.1%), and soft drinks (1.9%), and for children, plain milk (3.1%), flavoured milk (2.8%), and fruit juice (2.6%). Coffee (10.6%) made the greatest contribution to calcium intake for adults; and plain milk (9.9%) and flavoured milk (7.6%) for children. The greatest contributors to vitamin C intake were fruit juice (13.4%) and alcoholic drinks (6.1%) for adults; and fruit juice (23.4%) for children. For total sugar intake, soft drinks (8.0%), coffee (8.4%), and fruit juice (5.9%) made the highest contribution for adults; and fruit juice (9.8%) and soft drinks (8.7%) for children. The type and amount of beverage consumption has considerable relevance to dietary quality for Australians.

## 1. Introduction

Beverage intake is frequently included in food-based national dietary guidelines. These guidelines provide context-specific and scientifically based recommendations for a health-promoting diet, responding to the public health and nutrition priorities and population consumption patterns, among other things [1]. A recent review of food-based dietary guidelines for countries on the North and South American continents [2] indicated that 90% of these guidelines (27 of 30) included recommendations about beverages, and half recommend drinking at least 2 L of water a day. An earlier review found that 79% of European countries included recommendations about beverages [3]. Previous research suggests there is significant variation in average beverage intake in relation to both the type and amount consumed within and between countries [4,5,6]. Unsurprisingly, the differences between countries appears to be reflected in the availability of beverage types in local supermarkets [7]. 

The current Australian Dietary Guidelines [8] recommend drinking plenty of water, as well as reduced fat milk and occasionally fruit juice. These guidelines also provide advice on limiting alcohol consumption and drinks that contain added sugars and salt.

Beverages show a substantial diversity of energy, macronutrient, and micronutrient content [9], with some beverages containing specific bioactive constituents [10,11]. Comprehensive descriptive analyses of all beverage intake within a population are relatively infrequent and mainly oriented to assessing total water and energy intake [12,13,14,15] or association with other dietary components [16]. There has been some attention given to understanding categories of beverage intake such as sugar-sweetened beverages [17,18,19,20,21], sports and energy drinks [22,23], alcoholic beverages [24], 100% fruit juice [25,26], and dairy drinks [27]. This is probably a consequence of interest in particular nutrients or components within these categories; however, consideration of all beverages gives greater clarity to the role of beverages and insight into their nutritional contribution to total intake. 

The aim of this analysis was to examine the contribution of beverages and beverage sub-categories to total energy and selected nutrient intakes in the Australian population using data from the 2011–2012 National Nutrition and Physical Activity Survey.

## 2. Materials and Methods

### 2.1. The Australian National Nutrition and Physical Activity Survey

The Australian Health Survey (AHS) was conducted by the Australian Bureau of Statistics (ABS) in 2011–2013, reaching 32,000 people (25,000 households). The AHS is comprised of three components: the National Health Survey (NHS), the National Nutrition and Physical Activity Survey (NNPAS), and the National Health Measures Survey (NHMS). The secondary analysis conducted for this report utilised data collected from the 12,153 respondents completing the NNPAS.

A more detailed description of the sampling framework and data collection methods of the Australian Health Survey is available in the comprehensive ‘Users Guide’ accessible on the ABS website: http://www.abs.gov.au/ausstats/abs@.nsf/mf/4363.0.55.001.

Briefly, the method used to collect the dietary intake data as part of the NNPAS was two 5-phase, 24-h recalls where respondents were asked to recall the previous 24 h intake of food, beverages, and dietary supplements. The first recall was conducted face-to-face with a trained interviewer, and the second dietary recall was attempted with all respondents at least 8 days later via a telephone interview.

For children aged less than 15 years the interview was conducted primarily with a parent or guardian, and children were encouraged to participate. Parental consent was granted to interview respondents aged 15–17 years, while some parents opted to provide this information on the child’s behalf.

To improve accuracy and quality of the data collected, interviewers used an Automated Multiple-Pass Method (AMPA) developed by the United States Department of Agriculture and adapted by the ABS together with Food Standards Australia and New Zealand (FSANZ) to better reflect the Australian food supply. This method attempts to maximise respondents’ memory recall and was used in conjunction with a Food Model Booklet to assist respondents in the estimation of portion size and quantities of recalled items.

### 2.2. Secondary Analysis Methodology

All analyses were conducted using the first day of dietary recall (day 1 data) and excluded any nutrient intake originating from supplements. The second day of dietary recall was not used because only 64% of participants completed a second day recall. Results were predominantly grouped, and maintaining the integrity of one day of intake allows us to make valid comment on the range of intake on a day (i.e., the 10th to 90th percentile).

Permission to access the survey database and to conduct this secondary analysis was obtained from the ABS prior to commencing the work. 

The NNPAS dataset provides multiple lines of data, one for each food and beverage consumed at specific times across the day for each subject in the survey. Each food is identified by an 8-digit hierarchical food code that allows liquid foods to be distinguished by the first 3 digits of the code. A food combination code (combocode) identifies where separate food data lines were combined into a single food to be consumed (for example, milk and breakfast cereal into a single consumed food; coffee, sugar, and milk into a single consumed beverage). The first step of classifying beverage consumption was to identify and exclude liquids which were combined with other foods prior to consumption and consumed as a food, not beverage (for example wine added to a casserole). These were identified using the combocode and the specific time of consumption. The combocode was also used to re-join and correctly allocate liquids to a beverage category where more than one liquid was combined prior to consumption. For example, when vodka was mixed with orange juice, the combined beverage was categorised as an alcoholic beverage rather than fruit juice, and tea with added milk and sugar was combined and categorised as tea rather than plain milk or sugar.

Liquids that were not associated with a combocode were assumed to be consumed as a beverage. Each beverage including any additions was only accounted for once. There were 448 different beverages consumed by participants in the survey, each with an associated nutrient composition. All liquids were then categorised into a beverage category for analysis (Table 1).

Nutrient contribution was estimated from the individual beverage and aggregated for each participant into a beverage category or into beverages overall.

### 2.3. Statistical Analysis

Statistical analyses were performed using the IBM SPSS statistical software package version 23 (SPSS Inc., Chicago, IL, USA). Beverage consumption patterns were examined by gender (male, female, total) and by age (2–3, 4–8, 9–13, 14–18, 19–30, 31–50, 51–70, 71+ years), as well as aggregated groups of children aged 2–18 years; adults aged 19+ years, and total population (2+ years). For each beverage classification, estimates were calculated for the percent of the population group who consumed the beverage, the mean daily intake for all respondents who consumed any of the category on the surveyed day, and the 10th and 90th percentile of intake for the same group. If 20 subjects or less consumed from the beverage category for any demographic subgroup, no estimate for mean daily intake was calculated.

The contribution of beverages to total nutrient intake was estimated for energy intake, calcium, vitamin C, and total sugar intake, excluding nutrients from supplements. Estimates were calculated for consumers of each beverage category and for the total population within each demographic group.

Summary estimates were weighted to reflect the demographic structure of the Australian population using weights based on age, gender, and residential area that are included in the dataset by the Australian Bureau of Statistics. An additional weighting factor was applied to correct for the day of the week the survey was recorded for because the percentage of subjects reporting their intake for Saturday (3.5%) and to a lesser extent Friday (11.4%) were under-represented compared to the expected percentage of 14.3%. The population weights were rescaled to the size of the sample for inferential statistics and calculation of 95% confidence limits for mean estimates.

The difference between males and females for mean beverage intake was tested for statistical significance using the Mann–Whitney U-test with no adjustment for multiple comparisons. The statistical difference between age groups was tested following a Kruskal–Wallis test using post hoc comparisons with Bonferroni adjustment. A chi-squared test was used to assess difference of proportions between categories. *p*-Values of less than 0.05 were taken as indicating statistical significance.

## 3. Results

### 3.1. Assessment of Population Consumption

Almost all survey subjects (99.9%) reported consumption of one or more beverages during the day of the survey (Supplementary Table S1). The median daily number of beverage categories consumed per person was two for children and three for adults, and the 90th percentile for the number of daily beverage categories consumed was three for children and four for adults (data not shown). The median total beverage intake for the population was 1878 g, with the 90th percentile being 3551 g. The median total intake of beverages consumed was greater for adults than children (2061 g compared to 1275 g, *p* < 0.001) and greater for males compared to females (2003 g compared to 1760 g, *p* < 0.001). After water (consumed by 84% of adults and 90.5% of children), the beverage categories consumed by the highest percentage of adults were coffee (60.0%), tea (48.3%), alcoholic drinks (33.0%), and soft drinks (26.7%); and for children were fruit juice (38.7%), soft drinks (28.3%), plain milk (28.1%), and flavoured milk (20.8%). Energy drinks, other beverages, and milk alternatives were consumed by less than 5% of adults, and coffee, energy drinks, milk alternatives, and alcoholic drinks were consumed by less than 5% of children. A higher percentage of adult men than women consumed alcoholic beverages (41% compared to 26%; *p* < 0.001) and soft drinks (30% compared to 24%; *p* < 0.001), but a higher percentage of women consumed tea (55% compared to 41%; *p* < 0.001). Differences in the percentage of males and females consuming different beverage categories was less pronounced in children.

**Figure 1:**
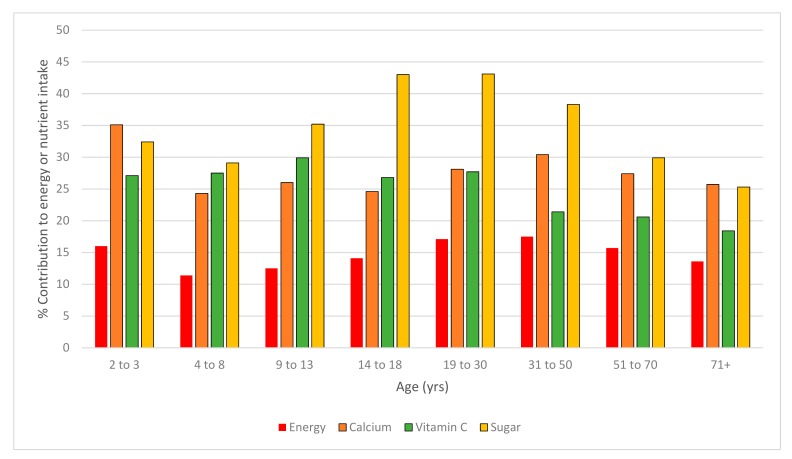
The percentage contribution of total beverage intake to total dietary energy, calcium, vitamin C, and sugar intake by age group.

**Figure 2:**
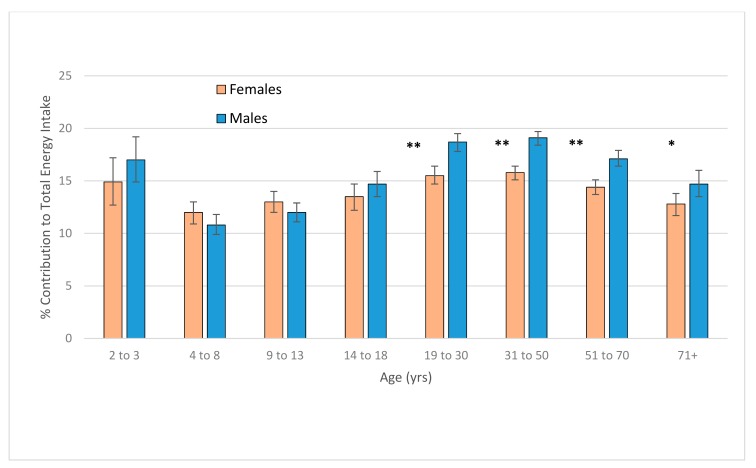
The percentage contribution (mean ± 95% confidence interval [CI]) of total beverage intake to total dietary energy intake for Australians by age group and sex. * Difference between males and females statistically significant at *p* = 0.01; ** Difference between males and females statistically significant at *p* < 0.001.

**Figure 3:**
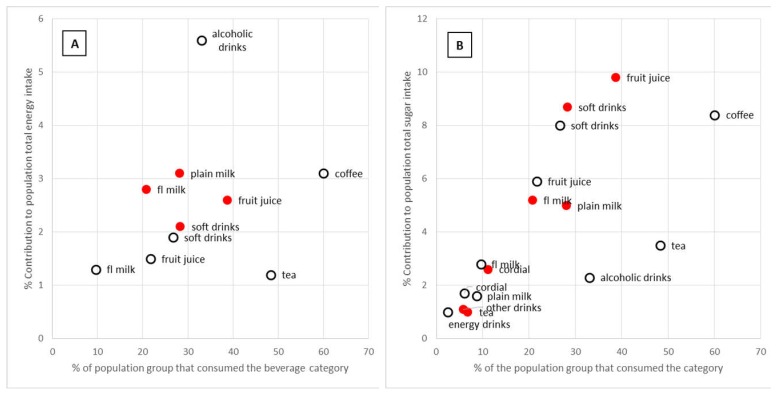
The contribution of beverage categories to (**A**) total dietary energy intake and (**B**) total sugar intake for Australian children (2 to 18 years, 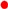) and adults (19 years and older, 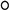) by the prevalence of consumption on the day of the survey. Only categories contributing 1% or more to the total energy or nutrient intake are shown.

Beverage intake accounted for 15.8% of energy on average across the population. The contribution of beverages to total energy was highest in children aged 2–3 years (16.0% of energy), and in the 19–30 and 31–50 year adult age groups (17.1% and 17.5% of energy, respectively). For every age group examined, the contribution of beverages to total energy exceeded 10% of total energy intake and was greater than 15% for all adults aged 19 to 70 years (Figure 1). Adult men, compared to adult women, consumed a significantly greater proportion of their energy intake as a beverage; this was consistent across all adult age groups (Figure 2). This was also consistent with adult men having a higher average daily amount of every beverage category compared to women except for tea (Supplementary Table S1). The beverage categories that made the greatest contribution to total dietary energy intake were alcoholic drinks (5.6%) and coffee (3.1%) for adults; and plain milk (3.1%), flavoured milk (2.8%), fruit juice (2.6%), and soft drinks (2.1%) for children (Figure 3).

The contribution of beverages to total dietary sugar intake (without distinction between natural or added sugars) is also shown in Figure 1. The largest contribution of beverages to dietary sugar intake was for the 14–18 years and 19–30 years age groups where beverage intake contributed 43% of the total dietary sugar intake. For all age groups, beverage intake contributed more than 25% of dietary sugar intake. The beverage categories that made the greatest contribution to total sugar intake were coffee (8.4%), soft drinks (8.0%), fruit juice (5.9%), tea (3.5%), flavoured milk (2.8%), and alcoholic drinks (2.3%) for adults; and fruit juice (9.8%), soft drinks (8.7%), flavoured milk (5.2%), plain milk (5%), and cordial (2.6%) for children (Figure 3).

**Figure 4:**
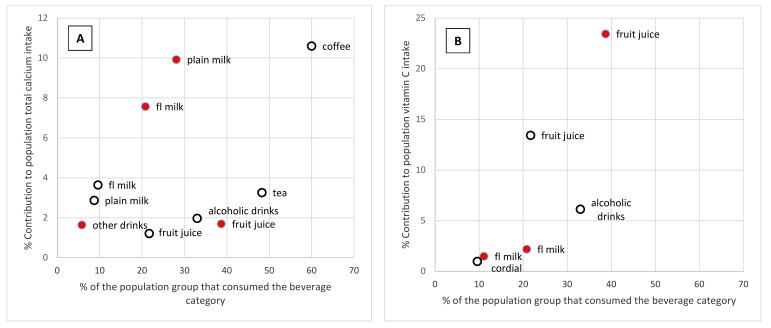
The contribution of beverage categories to (**A**) total dietary calcium intake and (**B**) total dietary vitamin C intake for Australian children (2 to 18 years, 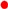) and adults (19 years and older, 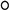) by the prevalence of consumption on the day of the survey. Only categories contributing 1% or more to the total nutrient intake are shown.

For every age group, the percentage contribution of beverages to calcium intake and vitamin C intake was greater than its contribution to energy intake (Figure 1). For children aged 2–3 years, beverages made the greatest contribution to calcium intake (35.1%), but the contribution of beverages to calcium intake was almost, or above, 25% for all age groups. The beverage categories that made the greatest contribution to calcium intake were coffee (10.6%), flavoured milk (3.6%), tea (3.3%), and plain milk (2.9%) for adults; and plain milk (9.9%) and flavoured milk (7.6%) for children (Figure 4).

The contribution of beverages to total vitamin C was above 20% for all age groups except the oldest adult age group (71 years and older). Beverages made the greatest contribution to vitamin C intake for children aged 9–13 years, where almost 30% of total vitamin C intake was from beverages. Beverage categories with the highest contribution to vitamin C intake were fruit juice (13.4%) and alcoholic drinks (6.1%) for adults; and fruit juice (23.4%) and flavoured milk (2.2%) for children (Figure 4).

### 3.2. Assessment of Consumption by Consumers Only

The contribution of a beverage category to total energy or nutrient intake of the population is related to its composition, the percentage of the population who consumed it, and the mean amount consumed (in addition to other dietary sources of energy or nutrient intake).When the dietary intake is assessed only for those persons who consumed a beverage from the category, the contribution of the beverage category to mean total intake is higher (Supplementary Table S2). For adults who consumed tea on the day of the survey, it contributed an average of 2.7% to their total energy intake, but 7.1% to their total calcium intake and 7.6% to their total sugar intake. For adults who consumed one or more alcoholic drinks, this category contributed a mean of 17.1% to total energy intake, 18.7% to total vitamin C intake, and 6.9% to total sugar intake. For children who consumed fruit juice on the day of the survey, it contributed 60.1% to their total vitamin C intake, 25.2% to their total sugar intake, but only 6.6% to their total energy intake. Similarly for children who consumed flavoured milk on the day of the survey, it contributed 39.7% to their total calcium intake, 27.4% to their total sugar intake, and only 14.5% to their total energy intake.

The mean daily intake for consumers of water (983 g for child consumers, 1302 g for adult consumers) and of alcoholic drinks (862 g for child consumers [mostly males aged 14–18 years]; 806 g for adult consumers) is much higher than other beverage categories ( Supplementary Table S1). For adult consumers, the mean daily intake of soft drinks, tea, coffee, and flavoured milk is between 400 g and 600 g, and for consumers of cordial and energy drinks, it was marginally higher (606 g to 633 g). The mean daily intake for adult consumers of fruit juice, milk, and milk alternatives is between 250 g and 400 g, with other beverages lower at 228 g. For child consumers, the mean daily intake of each of cordial, soft drink, flavoured milk, and tea is between 350 g and 500 g, with the mean daily intake of consumers for energy drinks being higher (541 g). The mean daily intake for child consumers of fruit juice, milk, coffee, and milk alternatives is between 250 g and 350 g; and with other beverages it is lower at 166 g. Most of the child consumers of coffee and energy drinks were aged 14–18 years, and there was a tendency for consumers of soft drinks and tea to be from older age categories (Supplementary Table S1).

## 4. Discussion

Beverages are an important component of the dietary intake of Australians, providing 13.0% of total dietary energy for children and 16.6% of energy for Australian adults. A high prevalence [28] of body fatness in Australian children aged 5 to 17 years (24.9%) and adults (67%) means that energy over-consumption is a general dietary concern and must include beverage intake. To date, there has been considerable attention on specific categories of beverages, such as sugar-sweetened beverages and water, to support specific public health campaigns, but to our knowledge, this is the first examination of the role of all beverages to daily intakes of energy and nutrients. While making a substantial contribution to energy and sugar intake, beverages also contributed more than 25% of the total intake of calcium and more than 23% of vitamin C for both adults and children. Beverages are included in population dietary guidelines in most countries around the world, so it is important that their use is understood, for both the positive and negative aspects, in the context of the overall dietary pattern.

Similar to food consumption, beverage consumption differed by age and gender. For adults, the most commonly consumed beverages other than water were coffee and tea, alcohol, and soft drinks. However, as a consequence of energy density and the volumes consumed, the contribution to total energy intake was highest for alcohol, followed by coffee and soft drinks. Notably, the percentage contribution of beverage intake to total energy intake was higher for adult men than women. The population prevalence of beverage consumption for men was higher by at least 5% than women for soft drinks and alcohol, while for women it was at least 5% higher for tea and water. Furthermore, the mean amount consumed by adult male consumers was at least 100 g higher for cordial, soft drinks, energy drinks, plain milk, alcohol, and water compared to women. Within categories, drink types consumed by men and women may have different caloric values—for example, women consumed white wine more than other alcoholic drinks while men consumed beer [24]—however, the differences in intake for the specific beverage categories between men and women are large. Almost all of the adult men and women had a beverage on the day of the survey, yet the contribution of beverages for women to their smaller total energy intake was at least two percentage points lower for all age groups.

Australian children most commonly consume fruit juice, soft drink, plain, and flavoured milks, and each category provided between 2–3% of children’s total energy. Fruit juice also provided 22–25% of children’s total vitamin C intake depending on age group and up to 60% of total vitamin C among fruit juice consumers. While vitamin C is not generally regarded as a nutrient at risk of deficiency in the Australian population, and non-beverage sources are readily available (fresh fruit and vegetables for example), less than 6% of the adult population consume fruits and vegetables in the amounts recommended [28]. Furthermore, a recent South Australian study [29] measured serum vitamin C levels in 149 hospitalized elderly patients with 76.5% having hypovitaminosis C. The authors suggested the results might be indicative of a high prevalence of vitamin C deficiency in the community and recommended community surveys. Consumption of fruit juice is more prevalent in children than adults. 

Calcium is a nutrient for which subgroups of the population are recognized to have intakes below recommendations. Not accounting for supplement intake, across age groups, 43–94% of Australian adults and 11–94% of children aged 4 years and above don’t meet the estimated average requirement (EAR) for calcium [30], therefore the contribution from beverages is important in the context of overall intake. Beverages provide almost one quarter or more of total calcium intake from food for each of the age groups. Unsurprisingly, milk and beverages containing milk made a strong contribution to calcium intake in adults and children. For adults, coffee contributed almost three times more to calcium intake than milk, flavoured milk, or tea (10.6% compared to 2.9%, 3.6%, and 3.3%, respectively); while for children, plain milk (9.9%) and flavoured milk (7.6%) were the major contributors. 

The percentage of Australian children consuming plain or flavoured milk as a beverage was similar—28% of children consumed plain milk and 21% consumed flavoured milk. Using a 2007 national survey of children aged 2–16 years [31], the percentage of Australian children who consumed plain and flavoured milk was 65% or more for plain and 10% or less for flavoured, depending on age. A critical methodological difference was that the earlier analysis examined dairy foods as any part of intake (not only beverages) and defined flavoured milk as pre-mixed flavoured milks only while categorising milk with flavouring added in the plain milk category. In the present survey, the prevalence of intake of flavoured milk (home prepared and commercial) as a drink is higher than that of plain milk as a drink for the age groups 9–13 years and 14–18 years. Children offered flavoured milk have a higher overall milk intake than children drinking plain milk only with consequent nutritional benefits [32]. While added sugar intake for children should be discouraged, offering non sugar-sweetened flavoured milk may be a useful strategy to increase dairy food and calcium intakes in older children who do not generally meet their dietary recommendation [31].

Policy development specifically to reduce sugar sweetened beverage intake has been called for by many investigators [33,34,35,36,37] to reduce sugar and energy intake, although it has been acknowledged that targeting only beverages is an inappropriate policy response [35]. The present analysis found the contribution of beverages to total sugar intake was greater than 25% for all age groups, peaking at more than 40% for the 14–18 year and the 19–30 year age groups. Soft drinks and fruit juice were strong contributors to sugar intake for both adults and children, with coffee also making a substantial contribution for adults (6.7%) and flavoured milk and plain milk for children (5.2% and 5.0% respectively). Analyses of free or added sugars [35,38] highlight the contribution from soft drinks and fruit juice but largely overlook the sugar contribution from coffee and milk-based drinks because the intrinsic sugar in milk is not defined as a free or added sugar. Soft drinks, while the highest ranking beverage category in dietary sugar contribution for adults (8%), rank third (at 1.9%) behind alcoholic drinks and coffee in contribution to dietary energy intake. To strengthen an effort at the individual or population level to decrease overall energy intake from beverages, it is rational to also take into consideration beverages other than sugar sweetened soft drinks.

Consistent with the Australian Dietary Guideline recommendation to drink “plenty of water”, the water category was consumed by the highest percentage of Australians and in the highest daily amount. Water was consumed by more than 85% of people on the surveyed day, and the average daily intake for consumers was 1227 g. This is not necessarily a universal finding. For example, a large Dutch cohort of men and women aged 55–69 years old [39] found that mean water intake was only 95 mL/day, and 53% of men and 43% of women stated that they did not consume water at all. In the United States, while beverages provide 76–83% (according to age group) of total water intake for adults, older adults have been identified as potentially not consuming enough water [40]. In France [41], water intake is associated with higher dietary quality and lower intake of other beverages, in particular tea and coffee (although alcohol intake was not included in the analysis). In a randomised controlled trial setting [42], those achieving a high water intake (as a beverage) also had a greater increase in fruit and vegetables, as well as a greater decrease in salty snacks, cakes, and cookies. An increased water intake has also been implicated as assisting with weight loss or weight maintenance [43,44]. An intervention to increase water intake would be relatively simple and low cost, although a large community-based randomised controlled trial showed a better result on weight loss when diet beverages were increased compared to water [45].

The Australian Dietary Guidelines also advise “if you choose to drink alcohol, limit intake”. These guidelines and Australian alcohol guidelines (www.alcohol.gov.au) state that alcoholic drinks are not recommended for children, and adults are recommended to have no more than 2 standard (alcoholic) drinks on any day to reduce the risk of harm from alcohol-related disease or injury over a lifetime. In other analysis of the same survey [24], the median alcohol intake of adult men consumers was 3.3 standard drinks on weekdays and 4.1 on weekend days. For adult women consumers, the median intake was 2.7 standard drinks on weekdays and 3.1 on weekend days. From this analysis, the 90th percentile of intake was more than 1.8 L of alcoholic beverage. Even allowing for misreporting of alcoholic beverage intake, most Australian drinkers of alcoholic beverages exceed the recommended alcohol intake guidelines.

Mean beverage intake and the mean intake of different drink types varies considerably between countries for both children and adults [4,5,6,46]. Some countries have a substantially lower contribution of beverages to total energy intake (for example, 6% in Italy for 2005–2006, 8% in France for 2009, and 12% in Spain for 2013) [46]. The regional diversity highlights that national beverage intake is probably readily modifiable.

Comparison of beverage intake of Australians over time is problematic due to the small number of national surveys and differences in survey methodologies. Other data are available, such as sales data. In Australia, sales trends of non-alcoholic, water-based, ready-to-drink beverages were examined for the period 1997–2011 [47]. Sugar-sweetened carbonated soft drink sales declined from 75.8 to 56.1 L per person between 1997 and 2011, whereas non-sugar carbonated soft drink purchases increased from 22.8 to 28.2 L per person. Sales of still water increased by 12.4 L per person. The analysis did not cover all beverage types, reflected sales data rather than intake, and excluded tap water intake, therefore it is unclear whether total volume of beverage intake in Australia has changed. However, it is consistent with a large survey in South Australia [48] indicating a decrease in soft drink consumption between 2008 and 2013 among adults. These data show the consumption of soft drinks by Australians may be declining, led by sugar-sweetened soft drinks. It is reasonable to conclude that overall beverage choices have changed for Australians over a relatively short time period, possibly in a health-promoting direction. It is therefore somewhat realistic to expect that the Australian population could undertake beverage substitution to beverages that are better for them or otherwise reduce their intake of undesirable beverages [49]. In fact, the pleasure resulting from beverage consumption may not depend on the amount consumed [50] or the type of beverage consumed; perceived health impact may be a motivator for beverage consumption on at least some occasions. 

A strength of this secondary analysis is that it is based on large sample national dietary survey data with a moderately high response rate, therefore minimising the effect of non-response bias. The results are weighted to reflect the Australian population but also for day of the week to adjust for the disproportionate representation of certain days of the week due to the practicalities of collecting survey data. This is important given differences in beverage intake have been observed between week days to week-end days (e.g., [12,24]). Another strength of this study is the inclusion of all beverage categories, as well as the identification and exclusion of liquids that are combined with other foods and consumed as a food (in the example of milk added to cereal). This seems a sensible approach, because foods that are consumed as drinks are cognitively, functionally, and perhaps different in their physiological effect to foods that are eaten. However, the reasons that all drinks have not been commonly examined may be because researchers have a specific interest in one or few beverage categories, the technical difficulties in distinguishing drinks from foods eaten in large dietary datasets, and the fact that alcoholic drinks may be considered a special category of food that should be considered separately. This approach to dietary analysis has allowed for a comprehensive understanding of the role of beverages in the Australian diet. Limitations of the present study warrant discussion. Dietary survey data using 24 h recall are likely to under-report true dietary intake based on analysis of energy requirement [51,52], and misreporting may occur differentially between different foods and between foods and drinks. One 24 h dietary recall was used to assess the usual group intake because it maximized the number of participants included (only 64% of participants completed a second 24-h recall, and by telephone). The reported individual intakes represent beverage intake on a day, not usual beverage intake; however, the mean of the individual intakes for groups is a good representation of mean usual intake [53]. For this analysis, we used a range of broad categorisations of beverages, and while we described intake across 12 categories, further categorisation would provide finer detail. The categorisation of drinks requires subjective choices to be made which may not suit every purpose. In this analysis, soft drinks were categorised separately to energy drinks because of an expected different pattern of intake [22,23], and fruit juices included diluted fruit juices as well as pure fruit juices. These categories provide more detail than some previous; however, other investigators may have made other choices.

## 5. Conclusions

Beverages make an important contribution to the dietary energy intake of Australians (16%) and contribute more than a third to total sugar intake. Beverages also contribute about a quarter to total calcium and vitamin C intakes. The most commonly consumed beverage category is water, which was consumed by more than 85% of Australians on the day of the survey. For adults, the highest contributors to energy intake were alcohol and coffee, while for children, plain and flavoured milk and fruit juice were the highest contributors. Judicious beverage choice should aim to maximise its nutrient contribution while (for many people) considering its energy contribution.

## Figures and Tables

**Table 1:** Description of beverage category membership.

Beverage Category	Description
Alcoholic beverages	All beverages that include any alcohol content. Mixers were included in the category, but any alcoholic beverage used as an ingredient in food was not included.
Tea	All home-brewed tea plus all additions (milk, sugar, water) were included.
Coffee	Hot coffee plus all additions (milk, sugar) were included. Cold coffee-flavoured milk beverages were categorised as flavoured milks.
Soft drink	All flavoured carbonated beverages whether sugar-sweetened or sweetened with other sweetening agents. Energy drinks were not included.
Cordial	All flavoured drinks made up with water from a concentrate.
Energy drinks	All electrolyte (‘sport’ drinks) and energy drinks.
Fruit juices	All fruit and vegetable juices (non-carbonated), regardless of their dilution. Includes infant drinks based on fruit or vegetables.
Plain milk	Plain white milk without flavouring or additives, regardless of fat content. Milk used as an ingredient for food is not included; milk as an ingredient of beverages was included in the respective categories.
Flavoured milk	All flavoured milk (hot or cold) whether as purchased or produced through adding powdered or liquid flavouring to milk.
Milk alternatives	Plain or flavoured dairy milk alternatives such as soy milk and nut milks. Not used in food or as an addition to other beverage category.
Other beverages	Powdered flavourings with water, probiotic drinks, breakfast cereal beverages.
Water	All water consumed as a drink but not included in any other beverage category. Includes carbonated and still water.

## Supplementary Materials

The following are available online at https://www.mdpi.com/2072-6643/11/6/1389/s1, Table S1: Mean beverage intake (g) and 10th, 90th percentile for those who consumed any of the beverage category on the day of the survey, Table S2: Percentage contribution to energy, calcium, vitamin C, and total sugar intake for those who consumed the beverage (i.e., on the day that food intake was measured) and for the population.
