# Supplementary material for: Drink Choice is Important: Beverages Make a Substantial Contribution to Energy, Sugar, Calcium and Vitamin C Intake among Australians
Source: Nutrients. 2019 Jun 20;11(6):1389. doi: 10.3390/nu11061389 (PMC6627926; doi:10.3390/nu11061389)
Supplement: Supplementary file 1 [file nutrients-11-01389-s001.pdf]

Supplementary Table 1: Mean beverage intake (g) and 10th, 90th percentile for those who consumed any of the beverage category on the day of the survey

	Males		Females		p	Total	
Age Group (yrs)	Mean[1] (g) (10th-90th %ile)	n[2] (%)	Mean[1] (g) (10th-90th %ile)	n[2] (%)	Mean (Male vs female)	Mean[1] (g) (10th-90th %ile)	n[2] (%)
**All Beverages**							
2-3	1062 (267-1910)	227 (99.6%)	932 (350-1530)	235 (99.6%)	0.037	999[a] (307-1651)	462 (99.6%)
4-8	1200 (510-2000)	395 (99.5%)	1121 (549-1763)	392 (100.0%)	0.055	1162[a] (538-1937)	787 (99.7%)
9-13	1543 (580-2505)	391 (99.7%)	1470 (574-2519)	395 (100.0%)	0.151	1506[b] (574-2504)	786 (99.9%)
14-18	2079 (785-3718)	402 (99.8%)	1630 (716-2665)	369 (100.0%)	<0.001	1867[c] (784-3273)	771 (99.9%)
2-18	1547 (586-2759)	1415 (99.6%)	1356 (574-2343)	1391 (99.9%)	<0.001	1454 (584-2531)	2806 (99.8%)
19-30	2715 (1015-4804)	739 (100.0%)	2034 (945-3183)	853 (100.0%)	<0.001	2383[d] (975-4000)	1592 (100.0%)
31-50	2619 (1091-4524)	1669 (100.0%)	2182 (1021-3533)	1895 (99.9%)	<0.001	2406[d] (1069-3950)	3564 (100.0%)
51-70	2252 (985-3750)	1338 (99.8%)	2086 (893-3426)	1565 (100.0%)	<0.001	2169[e] (940-3540)	2903 (99.9%)
71+	1723 (808-2873)	532 (99.8%)	1688 (715-2805)	744 (99.9%)	0.52	1703[c] (747-2807)	1276 (99.8%)
19+	2465 (1000-4222)	4278 (99.9%)	2065 (929-3374)	5057 (100.0%)	<0.001	2265 (968-3755)	9335 (99.9%)
Total	2258 (862-3909)	5693 (99.8%)	1910 (787-3235)	6448 (100.0%)	<0.001	2086 (826-3551)	12141 (99.9%)
**Tea**							
2-3	-[3]	-	-	-		-	-
4-8	-	-	-	-		205[a] (49-338)	30 (3.8%)
9-13	-	-	387 (186-1000)	36 (9.1%)		385[a,b] (150-1000)	55 (7.0%)
14-18	434 (50-625)	37 (9.2%)	346 (200-534)	59 (16.0%)	0.035	396[a] (200-584)	96 (12.4%)
2-18	394 (80-633)	72 (5.1%)	322 (185-530)	119 (8.5%)	0.042	356 (129-611)	191 (6.8%)
19-30	375 (229-660)	136 (18.4%)	441 (207-819)	293 (34.3%)	0.002	416[a] (221-785)	429 (26.9%)
31-50	511 (200-1000)	583 (34.9%)	534 (200-1032)	950 (50.1%)	0.31	524[b] (200-1004)	1533 (43.0%)
51-70	584 (207-1225)	687 (51.2%)	597 (181-1103)	1003 (64.1%)	0.59	591[c] (200-1155)	1690 (58.2%)
71+	515 (198-1130)	335 (62.9%)	574 (190-1136)	528 (70.9%)	0.066	550[b] (191-1128)	863 (67.5%)
19+	520 (202-1017)	1741 (40.7%)	546 (200-1044)	2774 (54.8%)	0.047	535 (200-1033)	4515 (48.3%)
Total	514 (200-1012)	1813 (31.8%)	537 (200-1031)	2893 (44.8%)	0.066	527 (200-1022)	4706 (38.7%)
**Coffee**							
2-3	-	-	-	-		-	-

4-8	-	-	-	-		-	-
9-13	-	-	-	-		-	-
14-18	301 (205-504)	53 (13.2%)	311 (146-635)	47 (12.7%)	0.71	305^{a} (160-582)	100 (13.0%)
2-18	307 (203-539)	57 (4.0%)	311 (127-635)	54 (3.9%)	0.88	308 (160-582)	111 (3.9%)
19-30	377 (200-702)	265 (35.9%)	363 (200-660)	320 (37.5%)	0.45	370^{a,b} (200-671)	585 (36.7%)
31-50	519 (200-936)	1071 (64.2%)	477 (200-881)	1144 (60.3%)	0.011	498^{c} (200-919)	2215 (62.1%)
51-70	512 (181-1035)	920 (68.6%)	481 (166-981)	1037 (66.3%)	0.12	497^{c} (174-1018)	1957 (67.3%)
71+	421 (180-758)	347 (65.1%)	399 (136-714)	498 (66.8%)	0.35	409^{b} (153-731)	845 (66.1%)
19+	487 (200-936)	2603 (60.8%)	453 (181-850)	2999 (59.3%)	0.001	470 (187-905)	5602 (60.0%)
Total	481 (200-924)	2660 (46.7%)	450 (181-842)	3053 (47.3%)	0.002	466 (187-890)	5713 (47.0%)
**Fruit Juice**							
2-3	345 (84-818)	101 (44.3%)	276 (94-505)	92 (39.0%)	0.16	313^{a} (84-629)	193 (41.6%)
4-8	303 (130-526)	169 (42.6%)	300 (131-512)	155 (39.5%)	0.12	301^{a} (130-520)	324 (41.1%)
9-13	348 (158-728)	152 (38.8%)	347 (208-693)	152 (38.5%)	0.98	347^{a,b} (185-725)	304 (38.6%)
14-18	466 (210-914)	137 (34.0%)	373 (208-686)	131 (35.5%)	0.002	414^{b,c} (208-745)	268 (34.7%)
2-18	356 (131-735)	559 (39.4%)	333 (150-565)	530 (38.1%)	0.09	344 (147-630)	1089 (38.7%)
19-30	476 (260-756)	218 (29.5%)	378 (156-732)	214 (25.1%)	0.001	433^{c} (208-756)	432 (27.1%)
31-50	443 (156-840)	359 (21.5%)	360 (158-645)	370 (19.5%)	<0.001	406^{c} (158-735)	729 (20.4%)
51-70	342 (156-618)	278 (20.7%)	350 (105-634)	298 (19.0%)	0.67	346^{a} (141-634)	576 (19.8%)
71+	303 (154-472)	120 (22.5%)	335 (146-737)	167 (22.4%)	0.32	322^{a} (147-616)	287 (22.5%)
19+	420 (158-749)	975 (22.8%)	360 (147-651)	1049 (20.7%)	<0.001	391 (156-733)	2024 (21.7%)
Total	400 (156-742)	1534 (26.9%)	350 (147-634)	1579 (24.5%)	<0.001	376 (156-728)	3113 (25.6%)
**Cordial**							
2-3	416 (73-972)	28 (12.3%)	429 (77-1649)	26 (11.0%)	0.94	422^{a,b} (78-1039)	54 (11.6%)
4-8	425 (148-816)	52 (13.1%)	384 (147-788)	35 (8.9%)	0.48	408^{a} (147-788)	87 (11.0%)
9-13	480 (210-735)	57 (14.5%)	379 (146-630)	40 (10.1%)	0.06	440^{a} (210-735)	97 (12.3%)
14-18	541 (290-1026)	42 (10.4%)	439 (354-687)	32 (8.7%)	0.09	504^{a,c} (301-922)	74 (9.6%)
2-18	469 (204-815)	179 (12.6%)	398 (147-756)	133 (9.6%)	0.05	441 (163-788)	312 (11.1%)
19-30	671 (239-1132)	65 (8.8%)	567 (258-1313)	60 (7.0%)	0.24	637^{b,c} (261-1114)	125 (7.9%)
31-50	795 (210-1890)	134 (8.0%)	549 (257-982)	105 (5.5%)	<0.001	698^{c} (210-1530)	239 (6.7%)
51-70	602 (203-1102)	84 (6.3%)	509 (180-1200)	72 (4.6%)	0.27	560^{a,c} (200-1078)	156 (5.4%)
71+	545 (46-1411)	27 (5.1%)	267 (134-723)	23 (3.1%)	0.03	446^{a,c} (85-1088)	50 (3.9%)

19+	700 (210-1530)	310 (7.2%)	527 (210-1050)	260 (5.1%)	<0.001	633 (210-1446)	570 (6.1%)
Total	624 (210-1320)	489 (8.6%)	483 (202-927)	393 (6.1%)	<0.001	569 (210-1061)	882 (7.3%)
**Soft drinks**							
2-3	-	-	-	-		194[a,b] (91-390)	32 (6.9%)
4-8	263 (104-390)	63 (15.9%)	279 (97-437)	81 (20.7%)	0.44	272[a] (104-390)	144 (18.3%)
9-13	430 (195-750)	140 (35.7%)	404 (195-780)	128 (32.4%)	0.40	418[b] (195-757)	268 (34.1%)
14-18	626 (208-1305)	202 (50.1%)	406 (198-624)	149 (40.4%)	<0.001	530[c] (208-1055)	351 (45.5%)
2-18	485 (160-1040)	421 (29.6%)	368 (156-624)	374 (26.9%)	<0.001	430 (160-754)	795 (28.3%)
19-30	610 (260-1060)	320 (43.3%)	503 (250-780)	297 (34.8%)	<0.001	562[c] (260-1016)	617 (38.8%)
31-50	589 (260-1125)	572 (34.3%)	487 (208-780)	519 (27.4%)	<0.001	547[c] (250-1011)	1091 (30.6%)
51-70	610 (208-1276)	318 (23.7%)	443 (200-750)	298 (19.0%)	<0.001	542[c] (208-1092)	616 (21.2%)
71+	459 (156-894)	76 (14.3%)	355 (156-632)	95 (12.8%)	0.061	408[b] (156-778)	171 (13.4%)
19+	596 (260-1125)	1286 (30.0%)	478 (208-780)	1209 (23.9%)	<0.001	545 (208-1014)	2495 (26.7%)
Total	572 (208-1102)	1707 (29.9%)	451 (200-757)	1583 (24.5%)	<0.001	519 (208-970)	3290 (27.1%)
**Energy drinks**							
2-3	-	-	-	-		-	-
4-8	-	-	-	-		-	-
9-13	-	-	-	-		-	-
14-18	573 (394-624)	27 (6.7%)	-	-		564[a] (263-624)	46 (6.0%)
2-18	567 (335-624)	33 (2.3%)	471 (260-853)	27 (1.9%)	0.11	541 (261-624)	60 (2.1%)
19-30	751 (264-1560)	78 (10.6%)	358 (210-575)	28 (3.3%)	<0.001	622[a] (252-1560)	106 (6.7%)
31-50	590 (174-1248)	80 (4.8%)	631 (233-1483)	19 (1.0%)	0.65	598[a] (203-1248)	99 (2.8%)
51-70	-	-	-	-		546[a] (252-1040)	24 (0.8%)
71+	-	-	-	-		-	-
19+	672 (263-1506)	177 (4.1%)	432 (210-624)	57 (1.1%)	<0.001	606 (251-1303)	234 (2.5%)
Total	650 (263-1303)	210 (3.7%)	440 (226-624)	84 (1.3%)	<0.001	593 (252-1248)	294 (2.4%)
**Oth beverages**							
2-3	-	-	-	-		91[a] (4-281)	22 (4.7%)
4-8	162 (8-258)	25 (6.3%)	-	-		156[a,b] (5-295)	45 (5.7%)
9-13	169 (8-266)	34 (8.7%)	128 (6-311)	21 (5.3%)	0.24	158[a,b] (8-266)	55 (7.0%)
14-18	243 (190-263)	21 (5.2%)	210 (13-258)	20 (5.4%)	0.20	224[a,b] (129-258)	41 (5.3%)
2-18	174 (8-266)	91 (6.4%)	155 (7-265)	72 (5.2%)	0.32	166 (8-265)	163 (5.8%)

19-30	231 (20-339)	29 (3.9%)	236 (12-618)	46 (5.4%)	0.84	234[b] (12-516)	75 (4.7%)
31-50	206 (7-258)	28 (1.7%)	216 (9-344)	54 (2.8%)	0.75	212[a,b] (9-270)	82 (2.3%)
51-70	251 (9-423)	25 (1.9%)	267 (11-905)	33 (2.1%)	0.88	259[b] (10-410)	58 (2.0%)
71+	-	-	-	-		178[a,b] (10-258)	31 (2.4%)
19+	227 (17-339)	94 (2.2%)	229 (10-531)	152 (3.0%)	0.94	228 (11-396)	246 (2.6%)
Total	203 (9-339)	185 (3.2%)	204 (10-408)	224 (3.5%)	0.96	203 (9-339)	409 (3.4%)
**Plain milk**							
2-3	345 (103-776)	124 (54.4%)	380 (103-743)	131 (55.5%)	0.39	361[a,c] (103-773)	255 (55.0%)
4-8	256 (128-464)	121 (30.5%)	286 (52-569)	130 (33.2%)	0.21	270[b] (93-517)	251 (31.8%)
9-13	357 (156-642)	87 (22.2%)	342 (198-623)	97 (24.6%)	0.60	348[c,d] (156-627)	184 (23.4%)
14-18	446 (256-740)	56 (13.9%)	438 (206-1021)	43 (11.7%)	0.90	442[a] (243-735)	99 (12.8%)
2-18	330 (127-624)	388 (27.3%)	344 (129-628)	401 (28.8%)	0.44	337 (129-624)	789 (28.1%)
19-30	463 (214-927)	92 (12.4%)	364 (31-680)	85 (10.0%)	0.005	431[a] (93-927)	177 (11.1%)
31-50	336 (58-607)	149 (8.9%)	231 (32-412)	133 (7.0%)	<0.001	288[b,d] (52-515)	282 (7.9%)
51-70	300 (30-519)	123 (9.2%)	186 (31-368)	119 (7.6%)	0.001	241[b] (31-464)	242 (8.3%)
71+	259 (23-746)	47 (8.8%)	252 (42-535)	64 (8.6%)	0.89	255[b] (31-535)	111 (8.7%)
19+	375 (62-773)	411 (9.6%)	256 (31-516)	401 (7.9%)	<0.001	322 (42-627)	812 (8.7%)
Total	356 (103-711)	799 (14.0%)	297 (53-603)	802 (12.4%)	<0.001	329 (73-624)	1601 (13.2%)
**Flavoured milk**							
2-3	368 (83-912)	38 (16.7%)	327 (167-593)	43 (18.2%)	0.55	346[a,b,c] (142-621)	81 (17.5%)
4-8	325 (153-530)	85 (21.4%)	274 (157-520)	75 (19.1%)	0.042	302[a] (156-529)	160 (20.3%)
9-13	398 (227-636)	97 (24.7%)	408 (211-726)	95 (24.1%)	0.76	403[b,c] (218-726)	192 (24.4%)
14-18	431 (265-764)	80 (19.9%)	479 (199-999)	73 (19.8%)	0.33	455[b,d] (210-875)	153 (19.8%)
2-18	381 (210-660)	300 (21.1%)	383 (170-674)	286 (20.5%)	0.98	382 (195-672)	586 (20.8%)
19-30	526 (312-795)	98 (13.3%)	479 (215-795)	107 (12.5%)	0.23	506[d] (268-795)	205 (12.9%)
31-50	544 (263-951)	177 (10.6%)	387 (215-617)	184 (9.7%)	<0.001	476[c,d] (223-795)	361 (10.1%)
51-70	464 (206-1011)	107 (8.0%)	378 (205-712)	112 (7.2%)	0.044	425[b,d] (206-743)	219 (7.5%)
71+	328 (59-768)	40 (7.5%)	374 (208-619)	69 (9.3%)	0.38	354[a,b] (127-661)	109 (8.5%)
19+	508 (261-874)	422 (9.9%)	412 (212-682)	472 (9.3%)	<0.001	466 (218-795)	894 (9.6%)
Total	463 (218-795)	722 (12.7%)	400 (210-679)	758 (11.8%)	<0.001	434 (212-742)	1480 (12.2%)
**Milk alts**							
2-3	-	-	-	-		-	-

4-8	-	-	-	-		-	-
9-13	-	-	-	-		-	-
14-18	-	-	-	-		-	-
2-18	-	-	-	-		283 (72-531)	32 (1.1%)
19-30	-	-	-	-		-	-
31-50	-	-	275 (33-746)	21 (1.1%)		307[a] (65-566)	35 (1.0%)
51-70	-	-	-	-		257[a] (82-475)	25 (0.9%)
71+	-	-	-	-		-	-
19+	349 (237-524)	24 (0.6%)	256 (57-361)	53 (1.0%)	0.006	287 (82-412)	77 (0.8%)
Total	347 (206-572)	41 (0.7%)	253 (72-361)	68 (1.1%)	0.002	286 (82-444)	109 (0.9%)
**Alcohol**							
2-3	-	-	-	-		-	
4-8	-	-	-	-		-	
9-13	-	-	-	-		-	
14-18	1022 (258-2318)	24 (6.0%)	-	-		961[a,b,c] (258-2318)	29 (3.8%)
2-18	911 (29-2318)	26 (1.8%)	-	-		862 (29-2318)	34 (1.2%)
19-30	1124 (253-2274)	238 (32.2%)	633 (29-1719)	156 (18.3%)	<0.001	962[a] (159-2000)	394 (24.7%)
31-50	1089 (248-2587)	672 (40.3%)	510 (139-1010)	493 (26.0%)	<0.001	867[a,b] (150-2000)	1165 (32.7%)
51-70	940 (249-1945)	617 (46.0%)	473 (70-974)	503 (32.1%)	<0.001	751[c] (149-1529)	1120 (38.5%)
71+	516 (89-1139)	225 (42.2%)	348 (71-750)	176 (23.6%)	<0.001	448[d] (75-994)	401 (31.4%)
19+	995 (238-2273)	1752 (40.9%)	500 (99-1010)	1328 (26.3%)	<0.001	806 (149-1818)	3080 (33.0%)
Total	993 (235-2273)	1778 (31.2%)	500 (99-1010)	1336 (20.7%)	<0.001	807 (149-1818)	3114 (25.6%)
**Water**							
2-3	647 (90-1370)	201 (88.2%)	568 (200-1000)	203 (86.0%)	0.13	608[a] (129-1000)	404 (87.1%)
4-8	817 (240-1500)	371 (93.5%)	781 (250-1425)	369 (94.1%)	0.37	800[a,b] (240-1500)	740 (93.8%)
9-13	1012 (250-2000)	363 (92.6%)	1021 (276-2000)	362 (91.6%)	0.84	1017[c] (250-2000)	725 (92.1%)
14-18	1448 (350-3000)	347 (86.1%)	1157 (350-2000)	329 (89.2%)	<0.001	1305[d] (350-2449)	676 (87.6%)
2-18	1026 (250-2000)	1282 (90.3%)	938 (250-2000)	1263 (90.7%)	0.003	983 (250-2000)	2545 (90.5%)
19-30	1581 (360-3000)	666 (90.1%)	1354 (400-2400)	758 (88.9%)	<0.001	1472[e] (360-3000)	1424 (89.4%)
31-50	1438 (360-2813)	1373 (82.3%)	1338 (360-2491)	1678 (88.5%)	0.004	1388[d] (360-2500)	3051 (85.6%)
51-70	1131 (250-2000)	972 (72.5%)	1171 (291-2000)	1364 (87.2%)	0.29	1152[f] (280-2000)	2336 (80.4%)
71+	895 (200-1800)	405 (76.0%)	912 (250-2000)	637 (85.5%)	0.73	905[b,c] (250-2000)	1042 (81.5%)

19+	1357 (330-2608)	3416 (79.8%)	1250 (350-2200)	4437 (87.7%)	<0.001	1302 (350-2434)	7853 (84.1%)
Total	1277 (290-2500)	4698 (82.4%)	1180 (310-2054)	5700 (88.4%)	<0.001	1227 (300-2250)	10398 (85.6%)

[1]weighted mean, [2]sample n, [3]Summary data is not provided when cell count ≤20 individuals

Supplementary Table 2: Percentage contribution to energy, calcium, vitamin C, and total sugar intake for those who consumed the beverage (i.e. on the day that food intake was measured) and for the population.

Age grp (yrs)	% of Energy Intake[1]		% of Calcium Intake		% of Vitamin C Intake		% of Total Sugar Intake	
	Consumers	Population	Consumers	Population	Consumers	Population	Consumers	Population
**All Beverages**								
2-3	16.1[b,d,e]	16.0[b,d,e]	35.2[e]	35.1[e]	27.2[a,b]	27.1[a,b]	32.5[a,b,c]	32.4[a,b,c]
4-8	11.4[a]	11.4[a]	24.4[a]	24.3[a]	27.6[a]	27.5[a]	29.1[b,f]	29.1[b,f]
9-13	12.5[a,b]	12.5[a,b]	26.1[a,b,d]	26.0[a,b,d]	30.0[a]	29.9[a]	35.3[c,e]	35.2[c,e]
14-18	14.1[b,c]	14.1[b,c]	24.6[a,b]	24.6[a,b]	26.8[a,b]	26.8[a,b]	43.0[d]	43.0[d]
2-18	13.1	13.0	26.2	26.2	28.1	28.1	35.4	35.4
19-30	17.1[d,e]	17.1[d,e]	28.1[c,d]	28.1[c,d]	27.7[a]	27.7[a]	43.1[d]	43.1[d]
31-50	17.5[e]	17.5[e]	30.4[e]	30.4[e]	21.4[b]	21.4[b]	38.3[e]	38.3[e]
51-70	15.7[c,d]	15.7[c,d]	27.4[c]	27.4[c]	20.6[b]	20.6[b]	29.9[a,b]	29.9[a,b]
71+	13.6[b]	13.6[b]	25.8[b,c]	25.7[b,c]	18.4[b]	18.4[b]	25.3[f]	25.3[f]
19+	16.6	16.6	28.5	28.5	22.4	22.4	35.8	35.8
Total	15.8	15.8	28.0	28.0	23.7	23.7	35.8	35.7
**Tea**								
2-3	-	-	-	-	-	-	-	-
4-8	3.4[a,b]	0.2[a]	6.5[a,b,c]	0.3[a]	<0.1[a,b]	<0.1[a]	8.6[a,b]	0.4[a]
9-13	5.6[b,c]	0.4[a]	14.5[a,b]	0.9[a,b]	<0.1[a,b]	<0.1[a]	14.8[b]	0.9[a,b]
14-18	4.2[b]	0.6[b]	10.2[b]	1.5[b]	0.1[a,b]	<0.1[a,b]	13.8[b]	2.0[b]
2-18	4.4	0.3	10.6	0.8	<0.1	<0.1	13.0	1.0
19-30	2.4[a]	0.7[c]	6.0[c]	1.7[c]	<0.1[a,b]	<0.1[b, c]	7.4[a]	2.1[c]
31-50	2.8[a]	1.2[d]	7.3[a,c]	3.1[d]	<0.1[a]	<0.1[c]	7.9[a]	3.4[d]

51-70	2.6[a]	1.5[e]	7.2[a,b]	4.2[e]	0.1[b]	0.1[d]	7.4[a]	4.3[e]
71+	2.7[a,c]	1.8[f]	7.6[a,c]	5.1[e]	0.1[a,b]	0.1[d]	7.2[a]	4.8[e]
19+	2.7	1.2	7.1	3.3	0.1	<0.1	7.6	3.5
Total	2.7	1.0	7.3	2.7	0.1	<0.1	7.8	2.9
**Coffee**								
2-3	-	-	-	-	-	-	-	-
4-8	-	-	-	-	-	-	-	-
9-13	-	-	-	-	-	-	-	-
14-18	4.6[a,b]	0.8[a]	14.5[a,c]	2.6[a]	0.1[a]	<0.1[a]	12.7[a,b,c,d]	2.3[a]
2-18	4.7	0.3	14.8	0.8	0.1	<0.1	13.0	0.7
19-30	5.3[a]	1.7[b]	19.8[a,b]	6.4[b]	0.6[b]	0.2[b]	15.3[b]	4.9[b]
31-50	6.3[a]	3.9[d]	20.9[b]	13.1[d]	0.9[b]	0.5[c]	17.1[c]	10.7[e]
51-70	4.9[b,c]	3.2[c,d]	17.1[c]	11.3[c,d]	0.5[b]	0.3[c]	13.5[d]	8.9[d]
71+	4.6[c]	2.9[c]	14.9[c]	9.3[c]	0.8[b]	0.5[c]	11.1[e]	7.0[c]
19+	5.5	3.1	18.9	10.6	0.7	0.4	15.0	8.4
Total	5.5	2.5	18.8	8.4	0.7	0.3	15.0	6.7
**Fruit Juice**								
2-3	7.0[a,b,c]	2.9[c]	3.2[a]	1.4[c,d]	54.5[a]	22.9[c,d]	25.5[a]	10.7[a]
4-8	6.8[b,c]	2.9[c]	3.9[a,b]	1.7[d]	54.8[a,b]	23.4[d]	24.0[a]	10.2[a]
9-13	6.3[a,b,c]	2.6[c]	4.5[b,c]	1.8[d]	61.5[b,c]	25.1[d]	24.0[a]	9.8[a]
14-18	6.7[b,c]	2.2[b]	5.3[c,d]	1.7[b,c]	68.2[d]	21.8[b,c]	28.2[b]	9.0[b]
2-18	6.6	2.6	4.3	1.7	60.1	23.4	25.2	9.8
19-30	7.7[c]	2.2[b]	5.7[d]	1.6[b]	64.1[c,d]	18.3[b]	27.9[c]	7.9[b]
31-50	7.0[a,b]	1.5[a]	5.3[b,c]	1.1[a]	58.6[a,b]	12.2[a]	26.8[a]	5.6[c]
51-70	6.0[a]	1.2[a]	5.2[d]	1.0[a]	57.6[a,b]	11.2[a]	25.8[a]	5.0[c]
71+	6.2[a,b,c]	1.4[a]	4.9[b,d]	1.1[a]	56.0[a,b]	12.4[a]	22.4[a]	4.9[c]
19+	6.9	1.5	5.4	1.2	59.8	13.4	26.4	5.9
Total	6.8	1.8	5.0	1.3	59.9	15.6	26.0	6.8
**Cordial**								
2-3	7.7	0.8[b,d]	2.0[a]	0.2[b,d]	11.8[a,b]	1.3[a,b,d]	23.0[a]	2.5[b,c,d]
4-8	7.3	0.9[d]	1.6[a]	0.2[d]	16.3[a,b]	2.0[e]	24.3[a]	2.9[d]
9-13	7.6	0.8[c,d]	1.9[a,b]	0.2[c,d]	13.3[a,b]	1.5[c,d,e]	26.4[a]	3.0[c,d]

14-18	6.6	0.5[b,c]	1.6[a,b]	0.1[b,c]	13.2[a,b]	1.1[b,d]	24.3[a]	2.0[b,c]
2-18	7.3	0.8	1.7	0.2	14.1	1.5	24.9	2.6
19-30	7.5	0.5[b]	1.8[a,b]	0.1[b]	16.7[a,b]	1.2[b,d]	26.3[a]	1.8[b]
31-50	9.1	0.6[b]	2.5[b]	0.2[b]	15.1[a]	1.0[b,c,d]	31.0[a]	2.0[b]
51-70	7.3	0.4[a,b]	2.0[a,b]	0.1[a,b]	10.2[b]	0.5[a,b]	25.8[a]	1.4[a,b]
71+	7.5	0.2[a]	1.5[a,b]	0.1[a]	8.0[a,b]	0.3[a]	24.6[a]	0.8[a]
19+	8.1	0.5	2.1	0.1	14.0	0.8	28.1	1.7
Total	7.8[2]	0.5	2.0	0.1	14.0	1.0	27.0[3]	1.9
**Soft drinks**								
2-3	4.2[a,b,c]	0.2[a]	0.7[a,b]	<0.1[a]	0.9	0.1	14.9[a,b,c]	0.8[a]
4-8	5.2[a,c,d]	1.2[c]	0.8[a]	0.2[b,c]	<0.1	<0.1	21.0[b,c]	4.8[c]
9-13	6.8[b,d]	2.3[d]	1.2[b,c]	0.4[d]	0.6	0.2	26.7[a,b]	8.9[d]
14-18	8.0[b]	3.7[e]	1.5[c,d]	0.7[e]	<0.1	<0.1	33.2[a]	15.4[e]
2-18	6.9	2.1	1.2	0.4	0.2	0.1	28.0	8.7
19-30	7.3[b,d]	3.1[d]	1.5[d,e]	0.6[e]	<0.1	<0.1	29.9[a]	12.9[d]
31-50	6.0[c]	1.9[c]	1.5[c,e]	0.5[d]	0.1	<0.1	27.3[b,d]	8.6[c]
51-70	5.1[a]	1.2[b]	1.6[d]	0.4[c]	<0.1	<0.1	21.9[c]	5.0[b]
71+	5.4[a,c]	0.7[a,b]	1.4[c,d]	0.2[a,b]	0.3	<0.1	20.3[c,d]	2.6[a,b]
19+	6.2	1.9	1.5	0.5	<0.1	<0.1	26.8	8.0
Total	6.4	1.9	1.4	0.4	0.1[†]	<0.1[†]	27.1	8.2
**Energy drinks**								
2-3	-	-	-	-	-	-	-	-
4-8	-	-	-	-	-	-	-	-
9-13	-	-	-	-	-	-	-	-
14-18	7.0[a]	0.5[c]	6.3[a,b]	0.4[c]	<0.1[a]	<0.1	27.8	1.9[c]
2-18	6.8	0.2	6.9	0.2	<0.1	<0.1	27.2	0.7
19-30	8.5[b]	0.6[c]	8.0[a]	0.5[c]	<0.1[a]	<0.1	37.1	2.5[c]
31-50	8.2[a,b]	0.2[b]	6.4[b]	0.2[b]	<0.1[a]	<0.1	31.3	0.8[b]
51-70	6.7[a,b]	<0.1[a]	5.9[a,b]	<0.1[a]	0.6[b]	<0.1	32.8	0.2[a]
71+	-	-	-	-	-	-	-	-
19+	8.2	0.2	7.3	0.2	<0.1	<0.1	34.6	1.0
Total	7.9	0.2	7.2	0.2	<0.1	<0.1[†]	33.0[†]	0.9

**Oth beverages**								
2-3	6.2[a]	0.4[b,c]	12.0[a]	0.8[b,c]	4.9	0.3[a,b]	15.6[a,b]	1.0[b,c]
4-8	7.0[a]	0.4[b]	23.4[a,b]	1.4[b]	4.3	0.2[b]	12.3[a,b]	0.7[b]
9-13	6.1[a]	0.5[c]	18.3[a,b]	1.6[c]	5.8	0.5[b]	13.0[a]	1.1[c]
14-18	12.4[b]	0.7[b,c]	39.4[c]	2.3[b,c]	5.7	0.3[a,b]	24.4[b]	1.4[b,c]
2-18	7.9	0.5	23.9	1.6	5.3	0.4	15.9	1.1
19-30	7.1[a]	0.4[b]	20.6[a,b]	1.3[b]	5.5	0.3[b]	16.6[a,b]	1.0[b]
31-50	8.2[a,b]	0.1[a]	25.1[b,c]	0.5[a]	3.3	0.1[a]	18.1[a,b]	0.3[a]
51-70	8.6[a,b]	0.1[a]	23.5[a,b,c]	0.4[a]	10.9	0.2[a,b]	18.5[a,b]	0.3[a]
71+	7.1[a,b]	0.1[a]	19.7[a,b]	0.4[a]	10.7	0.2[a,b]	18.9[a],[b]	0.4[a]
19+	7.6	0.2	22.1	0.6	6.2	0.2	17.4	0.5
Total	7.7	0.3	22.8	0.9	5.9‡	0.2	16.8	0.6
**Plain milk**								
2-3	16.3[d]	8.9[d]	43.2[d]	23.6[d]	1.1[a]	0.6[a,b,c]	24.1[e]	13.2[e]
4-8	9.8[c]	3.1[c]	31.9[a,c]	10.0[c]	0.5[a]	0.2[a,b]	16.1[a,b,c]	5.0[d]
9-13	9.9[c]	2.7[c]	35.2[b,c]	9.6[c]	0.8[a,b]	0.2[b]	16.5[b,d]	4.5[d]
14-18	11.3[c,d]	1.3[a,b]	41.6[c,d]	4.7[a,b]	0.3[a]	<0.1[a]	18.7[c,d,e]	2.1[a,c]
2-18	11.5	3.1	36.7	9.9	0.7	0.2	18.4	5.0
19-30	11.1[c]	1.4[b]	37.4[b,c,d]	4.8[b]	1.9[a,b]	0.3[a,b]	22.0[e]	2.8[b,c]
31-50	7.2[a,b]	0.6[a]	28.2[a]	2.2[a]	0.6[a,b]	0.1[a]	14.4[a,b]	1.2[a]
51-70	6.5[a]	0.5[a]	26.3[a]	2.1[a]	0.7[c]	0.1[c]	14.0[a]	1.1[a]
71+	8.9[b,c]	0.8[a,b]	29.8[a,b]	2.8[a,b]	0.8[b,c]	0.1[a,b,c]	14.6[a,b,c]	1.4[a,b]
19+	8.5	0.8	31.0	2.9	1.1	0.1	16.9	1.6
Total	9.9	1.3	33.6	4.4	0.9	0.1	17.6	2.3
**Flavoured milk**								
2-3	17.5[b]	2.4[b,c]	46.3[b]	6.3[b,c,d]	13.9[a]	1.9[b,c]	28.2[a,b,c]	3.8[b,c,d]
4-8	12.9[a,b]	2.6[c,d]	38.2[a,b]	7.7[d,e]	8.1[a,b]	1.6[c]	23.0[b]	4.6[d,e]
9-13	13.8[b]	3.0[d]	38.5[a,b]	8.4[e]	10.7[a,b]	2.3[c]	29.1[c]	6.4[e]
14-18	16.2[b]	2.8[c,d]	41.0[b]	7.0[d,e]	15.5[a,b]	2.6[c]	30.0[c]	5.1[d,e]
2-18	14.5	2.8	39.7	7.6	11.4	2.2	27.4	5.2
19-30	15.3[b]	1.9[b]	42.0[b]	5.2[c]	13.7[a,b]	1.7[b]	32.0[c]	4.0[c]
31-50	14.9[b]	1.5[b]	39.9[b]	3.9[b,c]	9.8[b]	1.0[b]	30.6[c]	3.0[b,c]

51-70	13.6[a,b]	0.8[a]	38.0[a,b]	2.1[a]	9.1[a,b]	0.5[a]	29.7[a]	1.7[a]
71+	12.1[a]	1.0[a,b]	33.0[a]	2.9[a,b]	6.7[b]	0.6[a,b]	22.9[a,b]	2.0[a,b]
19+	14.5	1.3	39.6	3.6	10.7	1.0	30.2	2.8
Total	14.5	1.6	39.6	4.5	10.9	1.2	29.1	3.3
**Milk alts**								
2-3	-	-	-	-	-	-	-	-
4-8	-	-	-	-	-	-	-	-
9-13	-	-	-	-	-	-	-	-
14-18	-	-	-	-	-	-	-	-
2-18	11.0	0.1	31.6	0.3	8.4	0.1	16.4	0.1
19-30	-	-	-	-	-	-	-	-
31-50	6.9[a]	0.1	22.0[a]	0.2	1.4[a]	<0.1	11.5[a]	0.1
51-70	8.1[a]	0.1	25.6[a]	0.2	4.0[a]	<0.1	12.8[a]	0.1
71+	-	-	-	-	-	-	-	-
19+	7.8	0.1	24.9	0.3	1.8	<0.1	11.5	0.1
Total	8.4	0.1	26.1	0.3	3.0	<0.1	12.4	0.1
**Alcohol**								
2-3	-	-	-	-	-	-	-	-
4-8	-	-	-	-	-	-	-	-
9-13	-	-	-	-	-	-	-	-
14-18	17.6[a,b]	0.9[a]	3.9[a,b,c]	0.2[a]	12.8[a,b]	0.7[a]	29.0[c]	1.6[a]
2-18	16.0	0.3	3.5	0.1	11.4	0.2	25.8	0.4
19-30	18.0[a,b]	4.4[b]	6.1[b,c]	1.5[b]	22.8[b]	5.5[b]	11.7[b]	2.8[b]
31-50	17.4[b]	5.9[c]	6.0[c]	2.0[c]	18.6[b]	6.3[c]	7.4[a]	2.5[c]
51-70	17.3[b]	6.7[d]	6.5[a]	2.5[d]	18.7[b]	7.2[d]	4.6[a,b]	1.8[d]
71+	14.0[a]	4.5[b]	4.1[b]	1.3[c]	11.4[a]	3.7[b,c]	3.6[a]	1.2[b,d]
19+	17.1	5.6	6.0	2.0	18.7	6.1	6.9	2.3
Total	17.1	4.4	6.0	1.5	18.6	4.8	7.2	1.9
**Water**								
2-3	<0.1	<0.1	2.0	1.8	<0.1	<0.1	<0.1	<0.1
4-8	<0.1	<0.1	2.6	2.4	<0.1	<0.1	<0.1	<0.1
9-13	<0.1	<0.1	3.0	2.8	0.1	0.1	<0.1	<0.1

14-18	<0.1	<0.1	3.8	3.2	0.1	0.1	<0.1	<0.1
2-18	<0.1	<0.1	3.0	2.7	<0.1	<0.1	<0.1	<0.1
19-30	0.1	0.1	4.4	3.9	0.1	0.1	0.2	0.1
31-50	0.1	0.1	3.9	3.4	0.2	0.1	0.1	0.1
51-70	<0.1	<0.1	3.7	3.0	<0.1	<0.1	<0.1	<0.1
71+	<0.1	<0.1	3.0	2.4	0.1	0.1	0.1	0.1
19+	0.1	0.1	3.9	3.3	0.1	0.1	0.1	0.1
Total	0.1	<0.1	3.7	3.2	0.1	0.1	0.1	0.1

[1]All percentages are population weighted, estimates with the same superscripts are not statistically significantly different. [2]no evidence for statistical difference between age categories
[3]hypothesis that age category estimates are from the same distribution is rejected ($p<0.05$) but no statistically significant difference for pairwise comparisons
